# Metagenomic Geolocation Using Read Signatures

**DOI:** 10.3389/fgene.2022.643592

**Published:** 2022-02-28

**Authors:** Timothy Chappell, Shlomo Geva, James M. Hogan, David Lovell, Andrew Trotman, Dimitri Perrin

**Affiliations:** ^1^ School of Computer Science, Faculty of Science, Queensland University of Technology, Brisbane, QLD, Australia; ^2^ Centre for Data Science, Queensland University of Technology, Brisbane, QLD, Australia; ^3^ Department of Computer Science, University of Otago, Dunedin, New Zealand

**Keywords:** metagenomics, k-mer, signatures, geolocation, classification

## Abstract

We present a novel approach to the Metagenomic Geolocation Challenge based on random projection of the sample reads from each location. This approach explores the direct use of k-mer composition to characterise samples so that we can avoid the computationally demanding step of aligning reads to available microbial reference sequences. Each variable-length read is converted into a fixed-length, k-mer-based read signature. Read signatures are then clustered into location signatures which provide a more compact characterisation of the reads at each location. Classification is then treated as a problem in ranked retrieval of locations, where signature similarity is used as a measure of similarity in microbial composition. We evaluate our approach using the CAMDA 2020 Challenge dataset and obtain promising results based on nearest neighbour classification. The main findings of this study are that k-mer representations carry sufficient information to reveal the origin of many of the CAMDA 2020 Challenge metagenomic samples, and that this reference-free approach can be achieved with much less computation than methods that need reads to be assigned to operational taxonomic units—advantages which become clear through comparison to previously published work on the CAMDA 2019 Challenge data.

## 1 Background

The CAMDA Metagenomic Geolocation Challenge concerns the “global genetic cartography of urban spaces”, based on “extensive sampling of mass-transit systems and other public areas across the globe.” In the 2020 Challenge, participants received 1065 metagenomic fastq sample files collected from sites in 23 cities across six continents. The challenge is to determine—ideally with high confidence—the location of 121 Mystery samples. We took this opportunity to apply, extend and evaluate the k-mer-based signature methods that were used by Chappell et al. (2018) to classify wound microbiome data, motivated by the potential of these methods to scale to the volume of metagenomic data that the scientific community is accumulating.

This approach to analysing CAMDA Challenge data is novel because it obviates the need to align sequences to reference genomes of organisms thought to be present at sampled locations. Analyses of previous Challenge datasets have relied on this initial alignment step to provide information about the relative abundance of different species or OTUs (e.g, Casimiro-Soriguer et al. (2019); Walker and Datta (2019)). This is a computationally demanding and time consuming process—Zhang et al. (2021) report “OTU calling with QIIME required on an average approximately 500 CPU hours” with the CAMDA 2019 Challenge data. We wanted to explore the extent to which k-mer composition alone could be informative of geolocation, and whether a suitable analysis pipeline could be developed to extract that information in a reasonable time. This work does not therefore present a systematic benchmarking study of a tightly defined task, although we do highlight the computational advantages of our approach in the performance comparisons of Section 3.3.

After filtering out reads likely to be of human origin, we characterise samples by embedding them within a real-valued vector space of moderate dimension. This allows us to create a library of compact representations of each sample at a variety of resolutions. Mystery samples may be processed in a similar fashion and rapidly compared with entries in the library to facilitate classification.

**FIGURE 1:**
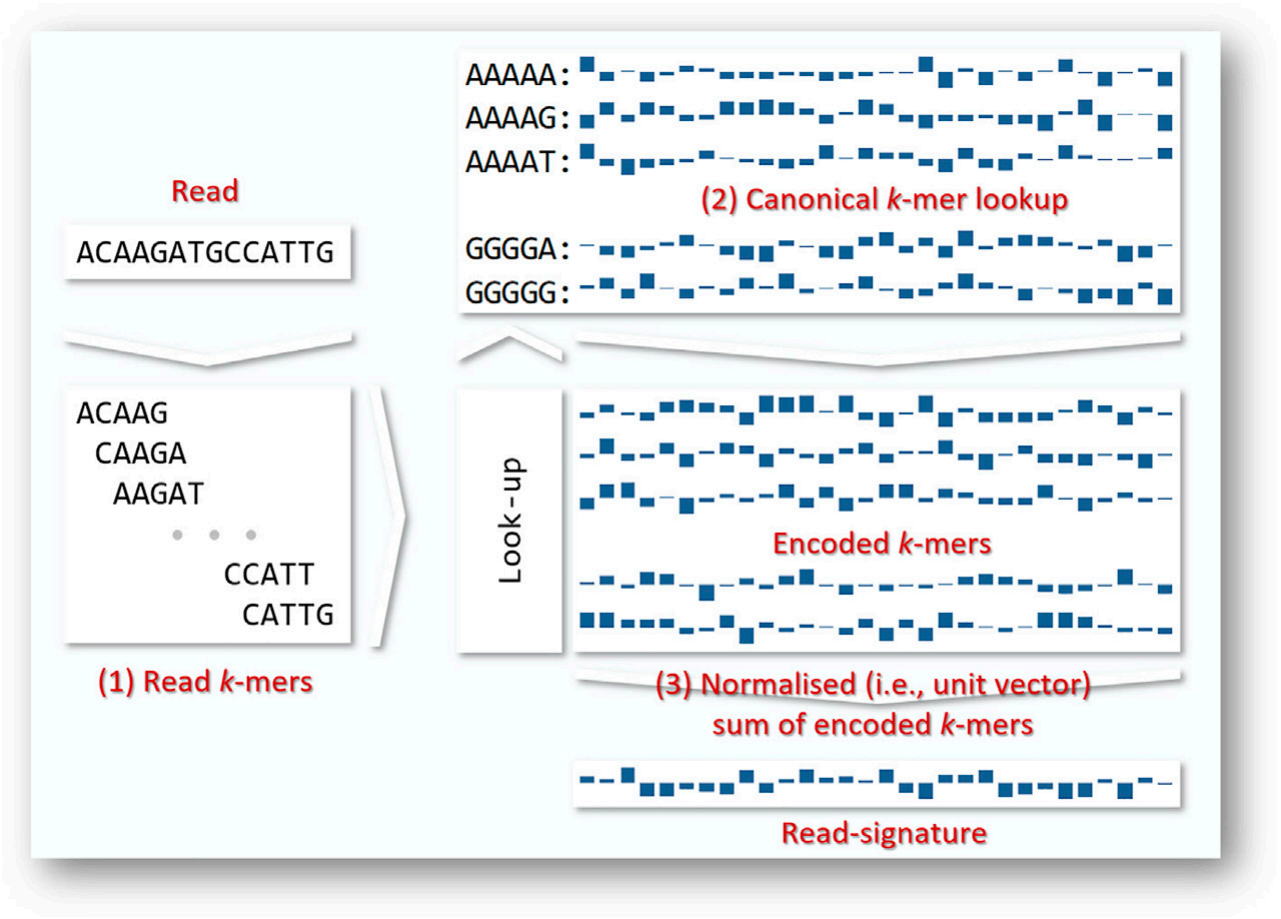
Merged reads vary in length from 50 to 292 bases. In general terms, we convert each read to a fixed-length read signature by (1) decomposing each read into its constituent read k-mers (2) using a look-up table to encode each of the read’s k-mers as a fixed length vector. A range of encoding strategies are possible; here we show encoding by random unit vectors. (3) the encoded k-mers are summed and normalised to a vector of unit length that we call a *read signature*. Since we do not know the direction of any given read, we work in terms of *canonical k-mers*, i.e., the k-mer or its reverse complement, whichever occurs first alphabetically.

Our approach encodes reads from each sample into signatures (Geva and De Vries, 2011), as illustrated in Figure 1. Each read is decomposed into its constituent k-mers, each of which corresponds to a unique random unit vector in $R^{N}$ . We sum the k-mer signature vectors from a given read, then divide by the magnitude of the sum to produce a unit-length read signature. This fixed-length signature vector representation affords more efficient comparison and clustering than the original sequences.

We apply hierarchical k-means clustering to the read signatures derived from each sample. This approach generates a tree of clusters, with the number of clusters increasing at each level. This allows us to represent the sample at a resolution of our choice. Each cluster may be represented by its mean and level—yielding a set of cluster-signatures which may be used for classification of novel samples. We evaluate the performance of our methods on samples and city labels from the 2020 Challenge dataset.

In the following sections, we describe the read datasets, and the process of read merging, filtering and transformation to the read signature representation (Sections 2.1–2.4). We explain how read signatures are clustered to produce location signatures for each city (Section 2.5) and outline our approach to classifying the Mystery samples (Section 2.6). Experiments with the Challenge datasets and the results obtained are described (Section 3) followed by discussion (Section 4), and conclusions (Section 5).

## 2 Materials and Methods

Source code to convert reads to read signatures, and to geolocate clustered signatures is available at github.com/tchappell/camda2020-code.

### 2.1 CAMDA Challenge Datasets

The main dataset used in this study combines the CAMDA Challenge collections *CSD16* and *CSD17*. We refer to this full collection as the *CAMDA 2020* dataset.

### 2.2 Initial Read Processing

CAMDA 2020 provided Illumina paired-end reads from 1065 location samples as training data. Some of the compressed files were partially corrupted, but 8.71 billion read pairs were successfully extracted. Read pairs were merged using PEAR v0.9.11 (Zhang et al., 2014) with default settings, except -p 1.0 to retain as much data as possible for analysis. PEAR merged 32.4*%* of the reads into 2.82 billion merged sequences. This collection was used in all further processing and analysis.

We decided to retain only the merged reads in this study to make the volume of data more manageable and because “By merging paired-end reads, the overlapping region between them can also be deployed for correcting sequencing errors and potentially yield sequences of higher quality” (Zhang et al., 2014).

### 2.3 Read Filtering

All CAMDA samples were specifically collected from locations where large numbers of people aggregate, such as mass transit systems and places of public gathering. With that in mind, we took the opportunity to explore the extent to which removing reads of human origin might improve our ability to discriminate locations, an approach used also by Walker and Datta (2019). We analysed three sets of sequence reads to explore the impact of eliminating human-related bacteria:

• all reads
• all non-human reads
• all non-human and non-*Cutibacterium acnes* reads.

We used Bowtie2 v2.4.1 (Langmead and Salzberg, 2012) to map the 2.82 billion merged sequences to the human genome, using the --sensitive-local settings and the *H. sapiens*, NCBI GRCh38 with 1KGenomes major SNPs pre-built index.

**FIGURE 2:**
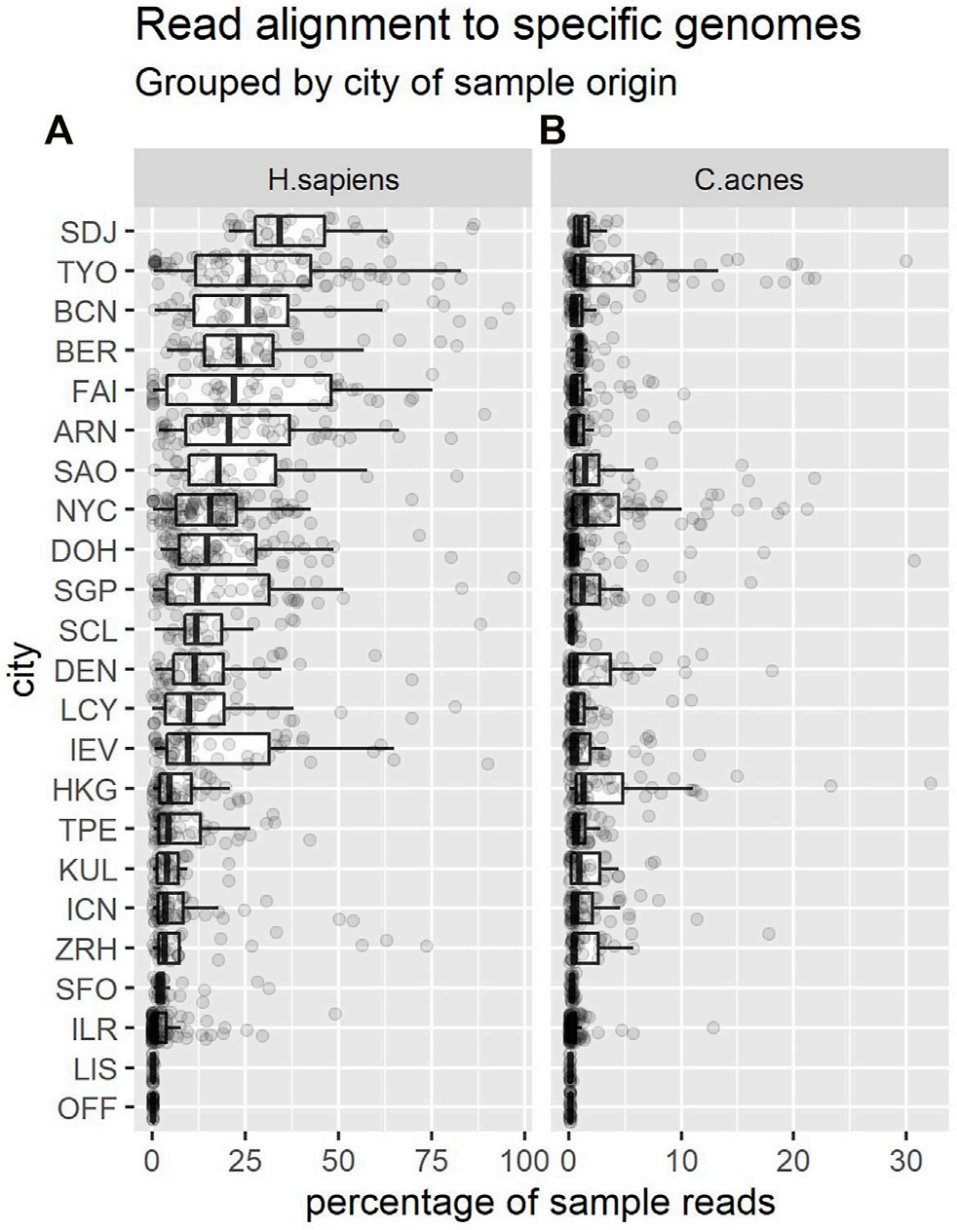
Each dot shows the percentage of reads from a sample that mapped to the *H. sapiens* genome **(A)** or *C. acnes* **(B)**. Each boxplot summarises the distribution of these proportions in samples from a given city. Cities are shown in descending order of median percentages for *H. sapiens*. Note the change of x-axis scale between panels.

Approximately 15.2*%* of CAMDA 2020 reads mapped to the human genome. The remaining 2.39 billion unmapped sequences were retained—this dataset is referred to as “nH” (for “non-human”). Figure 2 shows that the proportion of reads that mapped to the human genome varied markedly across cities.

We also decided to filter out *Cutibacterium acnes* reads, as these bacteria are common to human skin, relatively abundant, and therefore potentially unlikely to provide information discriminative of location. We used Bowtie2 as before, eliminating reads that mapped to the representative genome (*C. acnes* KPA171202) from NCBI. Approximately 1.4*%* of the nH sequences could also be mapped to the *C. acnes* genome. The remaining 2.36 billion unmapped sequences were retained, and this data is referred to as “nHnC” (for non-human and non-cutibacterium). Again, the proportion of reads mapping to this genome varied across cities (Figure 2).

### 2.4 Read Signature Transformation

Each of the 1065 CAMDA 2020 location samples is represented by a real-valued vector called a *signature* (Geva and De Vries, 2011) using the process described below. Signatures support compact representation, rapid clustering and comparison, allowing the creation of a library of signatures from known locations. Signature representations are obtained in the same way for each unseen or *Mystery* sample, and their origin is characterised—at least in principle—through comparison with known entries in the signature library.

We start by considering the representation of reads in a sample collection. A Collection of *M* variable-length DNA reads is available, and we represent them as a matrix of *M* fixed length signature vectors in $R^{N}$ using random projection (Johnson and Lindenstrauss, 1984).

As illustrated in Figure 1, we begin by extracting from each read its constituent k-mers. For each k-mer, we create a random signature in $R^{N}$ , and then combine these k-mer level signatures to yield the read signature. The value of *N* is arbitrary, and the desire for dimensionality reduction must be tempered by the need for representation capacity. For simplicity in the feature set, we work with the pure nucleotide alphabet {*A*, *C*, *G*, *T*}, discarding any k-mers which contain degenerate base symbols. If *k* is the chosen k-mer length, the cardinality of the set of all possible k-mers is then 4^*k*^, and for low values of *k* we will expect to see each of these k-mers appear in the collection. In these experiments we have opted to work with 2 ≤ *k* ≤ 6, based on earlier experience with large collections. There is a trade-off between storage requirements, representational capacity and speed of processing. It is likely that longer k-mers will prove more discriminative in small sets (Bernard et al. (2017)), but these advantages come with significant computational and storage overheads.

The k-mer signatures can in general be chosen at random. For large *N*, random vectors $x\in R^{N}$ tend to be nearly orthogonal. Here it is desirable to have k-mer-signatures that are mutually orthogonal, or at least approximately mutually orthogonal if the number of distinct k-mers is larger than *N*.

As noted above, *read-signatures* are generated by combining the underlying *k-mer-signatures*. For instance, with *k* = 4, we begin by initialising a matrix of k-mer-signatures of dimension 4^*k*^ × *N*, in this case 256 × 136, where 136 is the number of canonical 4-mers. The canonical kmers and the choice of *N*, the signature dimensionality, are explained below. Each read-signature is initialised to a vector of *N* zeros and we successively include k-mer-signatures by sliding a window of length *k* over the read, extracting the k-mer, looking up its signature, and summing with the current read-signature vector.

After applying this process to all of the reads in the sample, we obtain a matrix of *M* rows (the number of read-signatures) by *N* columns (the dimension of the vectors). The matrix is further processed to take account of the statistical properties of the collection. We control for k-mer frequency by subtracting the matrix column-wise mean from each row and we normalise so that each signature becomes a unit vector, removing the effect of variations in read length.

In the end we obtain the desired representation of the reads as a matrix of normalised read-signatures in $R^{N}$ . The process relies on a well-known property of random projections (Johnson and Lindenstrauss, 1984). Put simply, the random projection preserves mutual distance relationships between objects in the original space—if two objects are proximal, then their associated signature vectors will, with high probability, lie proximal in the projected space. A rigorous treatment of this property, and its validity in this context, is outside the scope of this paper. However, the experimental results here and elsewhere demonstrate its utility in text processing and bioinformatics domains (Chappell et al., 2018).

Since the reads are not aligned to a reference, we choose to work with canonical k-mers, regarding each k-mer and its reverse complement as identical entries. For instance, when using 4-mers, there are 136 canonical k-mers, and each k-mer and its reverse complement are assigned the same signature. This ensures that the projection is also canonical—a read and its reverse complement will be transformed to the same signature vector under this projection. In this case we project the reads onto $R^{136}$ , thus converting the collection of reads of variable length—ranging from around 125 to 300 base-pairs, with a mean of about 240—to a fixed dimensional space. The treatment is analogous for other choices of *k*.

### 2.5 Sample Clustering

Working with a sample represented as a matrix of read-signatures remains computationally expensive even if dimensionality reduction is applied. The number of reads is not reduced at all by the random projection process and each CAMDA sample may include millions of reads. Moreover, we have 1065 such samples. Using our pipeline, after assembling paired reads (with significant reduction and some loss as described above), there were 2.82 billion reads remaining. Even after filtering to remove reads likely of human origin, there are nearly 2.4 billion reads available. We thus use clustering over the read-signatures at the sample level to reduce the size of the representation and allow more efficient classification.

Tree-structured Vector Quantisation (TSVQ) is a recursive hierarchical k-means clustering approach based on the Euclidean distance between the vectors (Gersho and Gray, 1992). We apply it to each of the 1065 samples in turn. The process begins with the application of k-means clustering to the entire sample, and proceeds with the recursive application of k-means to each of the resulting k partitions. The process terminates when a specified tree depth is reached or the data are exhausted.

It is useful here to explore our intuitions of cluster membership and the relationship between cluster-signatures—the mean of the read-signatures within a cluster—and the original reads in the sample.

Clusters obtained via TSVQ are groups of nearby points in signature space. As the projections are locality-preserving, we expect that similar reads will share similar DNA. But unlike individual read-signatures, the resulting cluster-signatures cannot be traced back to any particular read (they represent a collection of similar reads). Clusters and their more compact cluster-signature vectors provide a sketch of the reads that are indirectly referenced by cluster membership. Of course the root is a sketch of the entire sample, in essence a sketch of the sketches below it, and so on. Since TSVQ cluster membership diminishes as we descend through tree branches, the sketches become more specific toward the leaves, and less specific towards the root. Working at a particular tree level allows us to choose a more specific or less specific representation of a sample, and indeed the entire CAMDA collection, depending upon the desired resolution.

### 2.6 Sample Classification

In order to classify each Mystery sample we apply simple Nearest Neighbour classification. Clusters obtained from the Mystery sample are compared with clusters from the labelled samples in order to make a prediction. The process is as follows:

1. Pre-process the mystery sample: paired read assembly, additional human-origin filtering, signature generation.
2. Use TSVQ to cluster the Mystery sample.
3. Find the nearest neighbour (NN) cluster of each Mystery cluster in the labelled collection.
4. Predict the location of each Mystery cluster as that of its NN cluster in the labelled collection.
5. Accumulate the cluster predictions. The final classification is the city associated with the location having the most clusters appearing as NN of clusters in the Mystery sample.

An extension of this basic approach is to use KNN (k nearest neighbours) in step 3, and in steps 4 and 5 to use all of the KNN clusters. We have experimented with this variation, using KNN, and accumulating the weighted vote of the KNN - each neighbour contributes a vote of 1/*rank* towards the classification, where *rank* is the neighbourhood rank position of each of the nearest neighbours.

## 3 Experiments and Results

### 3.1 CAMDA Challenge Training Data

To evaluate the performance of the classifier we performed leave-one-out cross-validation (LOOXV) over the samples. Each of the 1065 location samples is left out in turn, and a City prediction is made for that sample based on the most similar location sample. We thus score the classifier according to the accuracy of these predictions over the set of samples:

$$A=\frac{\text{Number of correctly classified samples}}{\text{Number of samples}}$$ (1)

The results below consider the 2020 collection in its merged (Section 2.2) and its merged and filtered (Section 2.3) forms. In each case, we run the LOOXV test over the entire collection. After paired-read assembly, the collection size was reduced from about 8.7 billion pairs to about 2.8 billion merged reads, a reduction of nearly 68*%*. Additional filtering reduces the size to 2.4 billion reads, an overall reduction of around 72*%*. We then converted 1 million reads from each sample into signatures (if the sample contained fewer than 1 million reads, all reads were used), resulting in a collection of 920 million signatures.

The signatures in each of the samples were then clustered by TSVQ using a tree order of 10, and tree depth of 4. We consider classification as described in Section 2.6 at three levels of depth in the tree.

In the case of the 2020 dataset, Level 1 or the root level consists of 1065 sample cluster-signatures; each sample is represented by a single signature. Level 2 represents each of the samples by 10 cluster-signatures, for a collection total of 10 ,650 signatures, and Level 3 represents each sample by 100 cluster-signatures, for a collection total of 100 650 signatures.

**FIGURE 3:**
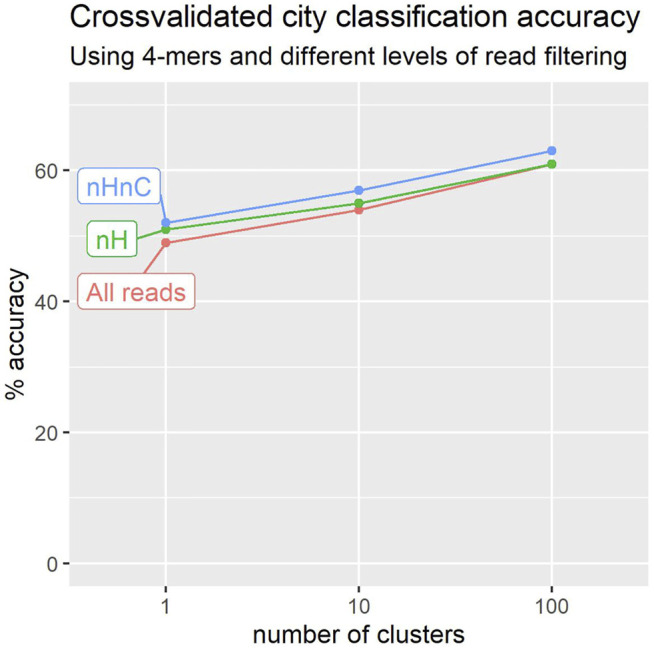
Leave-one-out crossvalidation suggests that we can more accurately predict a sample’s city of origin using reads that do not map to *H. sapiens* or *C. acnes* genomes (nHnC). Accuracy also improves with the number of location signatures used to characterise each city.

Figure 3 shows the results of these experiments over the 2020 collection (23 cities and 1065 locations). We experimented with 4-mers and three levels in the cluster tree, having 1 cluster, 10 clusters, and 100 clusters as described above. The best performance was obtained in both cases for the third level of 100 cluster-signatures. Surprisingly, a figure of 43*%* is obtained even when working with a single cluster per location, notwithstanding the 23 alternatives available. Even a single cluster-signature may capture the broader characteristics of a location sample.

Filtering to remove reads identifiable as of human origin yields a noticeable improvement in classification performance, with accuracy as much as 12*%* higher than that obtained prior to filtering (Figure 3).

**FIGURE 4:**
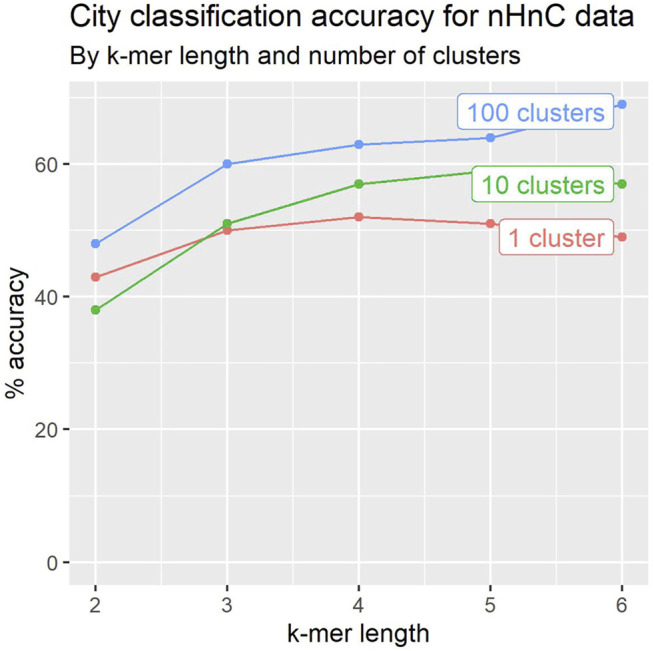
Leave-one-out crossvalidation accuracy for city classification tends to improve with k-mer length and number of location signatures.

To evaluate the impact of k-mer length, we experimented with 2 ≤ *k* ≤ 6. Figure 4 depicts the results for the 2020 dataset. The accuracy of the classifiers generally improves with k-mer length.

**FIGURE 5:**
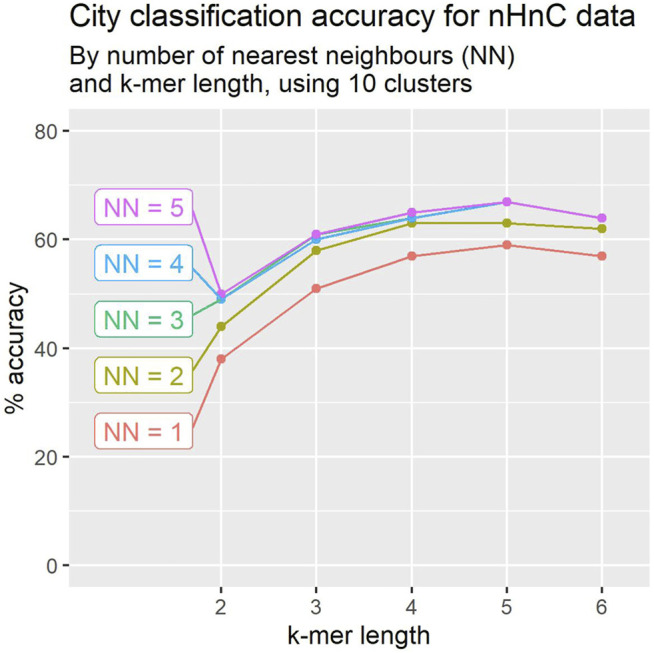
Using clusters of 10 location signatures, leave-one-out crossvalidation accuracy for city classification tends to improve with the number of nearest neighbours. Accuracy appears to peak for k-mer of length 5 in most cases.

In Section 2.6 we describe a modification to the algorithm, using KNN (k-nearest neighbours) to determine the class of a mystery sample. We worked with 10 clusters per sample, and varied the k-mer size and the number of neighbours. The results are depicted in Figure 5. The performance improves as more neighbours are considered, but improvements saturate at 3-NN. The performance improvement is highly significant across all k-mer size choices, with increases ranging between 8*%* and 11*%*.

It is useful to further assess the quality of prediction, taking into account that not all incorrect predictions are equal. If a ranked list of location predictions is produced, in a manner similar to a search engine, then it is useful to consider *S@N* (success at *N*), the frequency of a correct prediction appearing up to a given rank position *N*. For instance, *S@*1 = *A* (Eq. 1), and *S@*3 is the fraction of correct predictions appearing in any position from 1 to 3.

Here we are focused on the prediction of the city associated with the individual sample, rather than the identification of the city from a set of samples. This is a higher resolution view than the city view. Our approach is to rank all samples in the collection based on the number of cluster matches with the left-out sample.

As we classify each left-out sample, we keep track of the position at which the highest ranked “correct” location—from the same city as the left out sample—appears. The fraction of times that the first-ranked location matches the city of the left-out sample is the overall accuracy *A* in Eq. 1, the fraction of true positive predictions at rank 1. We also calculate *S@*2, the fraction of times that either the first or the second ranked location is from the correct city. This is done for all ranks; there are 1065 locations, and the desired behaviour is that, by analogy with a search engine, the *correct* locations—the same city as the left-out sample—are ranked as highly as possible: a location from the same city as the left out sample should preferably be ranked at or near the top of the list.

**FIGURE 6:**
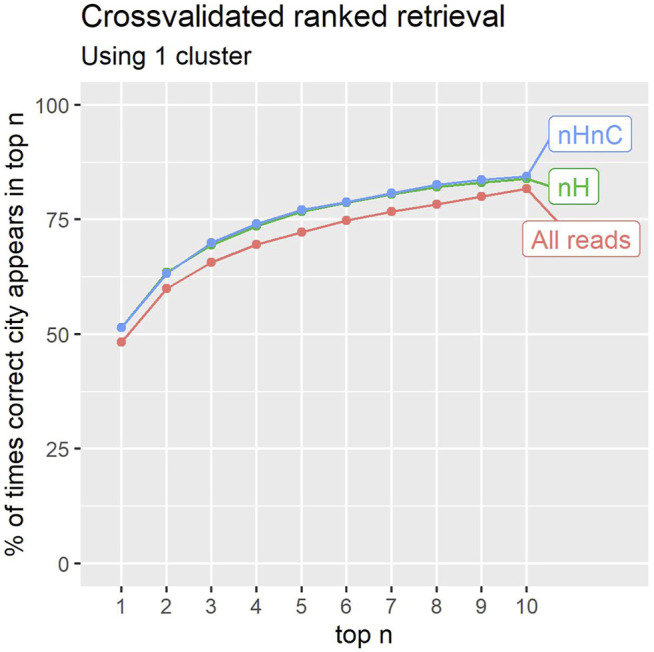
The percentage of times the correct city of a sample was identified within the first 1–10 results (counting ties). Retaining only non-human (nH), or non-human and non-*C.acnes* (nHnC) reads consistently improves retrieval.

The results shown in Figure 6 correspond to a classifier with 1 cluster per location. Results are reported for the unfiltered reads, the non-human reads and the non-human, non-*C.acnes* reads. The figure reports the precision obtained up to a given rank in the prediction list. In 84*%* of cases for the nHnC samples, a location from the same city is found in the 10 highest ranked location samples (from 1065 available). In an application involving further analysis of potential sources for a Mystery sample, this may prove useful.

### 3.2 CAMDA Challenge Mystery Samples

The prediction of the Mystery samples followed exactly the same process as we have used with cross-validation, and as described in Section 2.6. The results were compared with the ground truth mystery sample locations. There were five locations that were previously used and for which other samples appear in the CAMDA 2020 data set: HKG, IEV, TPE, TYO and ZRH. Another five new locations were added: Bogota, Krakow, Marseilles, Naples, and Vienna. However there is no reasonable way to distinguish those locations without additional information beyond the DNA samples. Therefore, we can only verify that our cross-validation predictions over the CAMDA 2020 training data are not a result of over-fitting.

**FIGURE 7:**
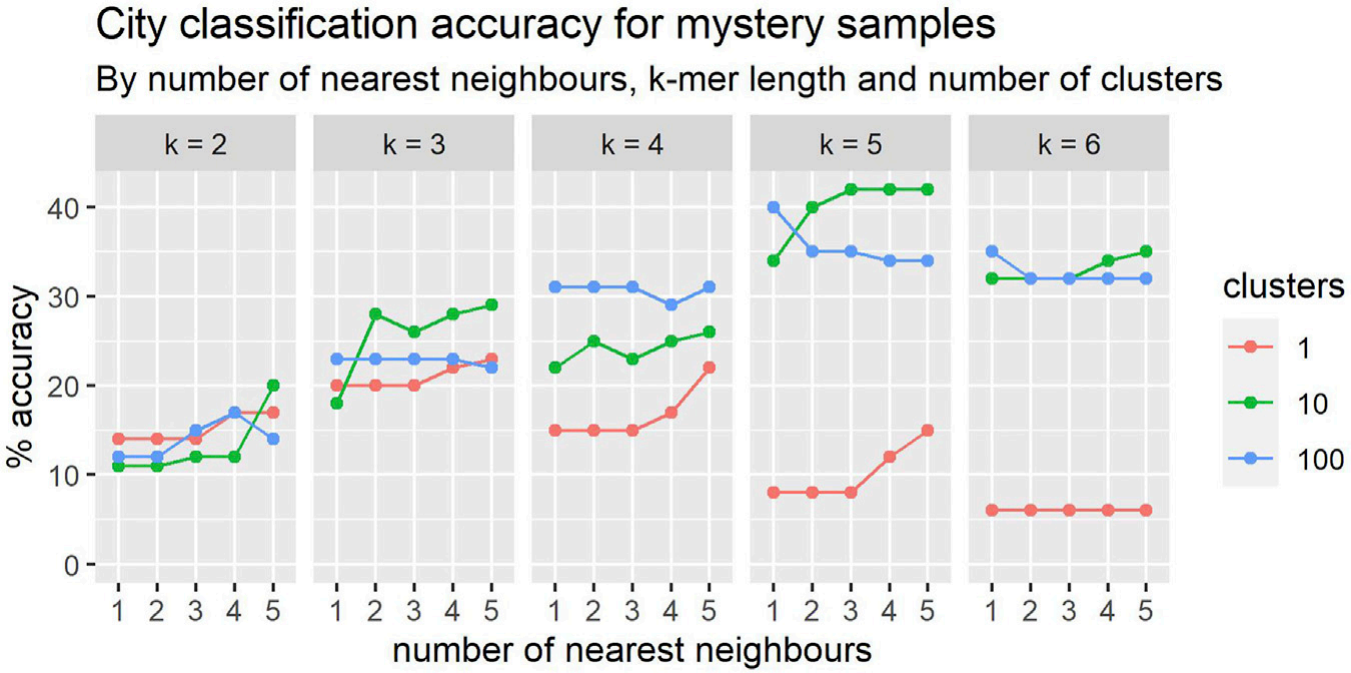
In general, the classification accuracy of mystery samples improves with the number nearest neighbours. The relationship between accuracy and k-mer length is more complex and there appears to be an interaction with cluster size.

The prediction accuracies over the set of five previously used cities appear in Figure 7. These results are in line with those obtained in the cross-validation studies over these same five cities, also being 30% with 4-mers.

### 3.3 Classification Performance Comparison

We compared our approach to the results of Zhang et al. (2021) who tackled a CAMDA challenge task of sample-based geolocation and gave the most detailed evaluation and performance information of the papers using recent challenge datasets.

To ensure fair and meaningful comparison, we re-ran our approach on the 2019 CAMDA challenge data that used, applied the same leave-one-out cross-validation performance estimates, and used all of the non-mystery samples except for two samples where only forward reads were present, resulting in a total of 300 samples, the same number reported by Zhang *et al.*

Our system was treated as a “black box” for the purposes of this comparison; we used the same filtering (removal of non-human non-Cutibacterium acnes reads) and the same approach for clustering and nearest neighbour classification. We used 10 clusters per signature and a *k*-mer length of 5. The approach was not tuned any further.

**FIGURE 8:**
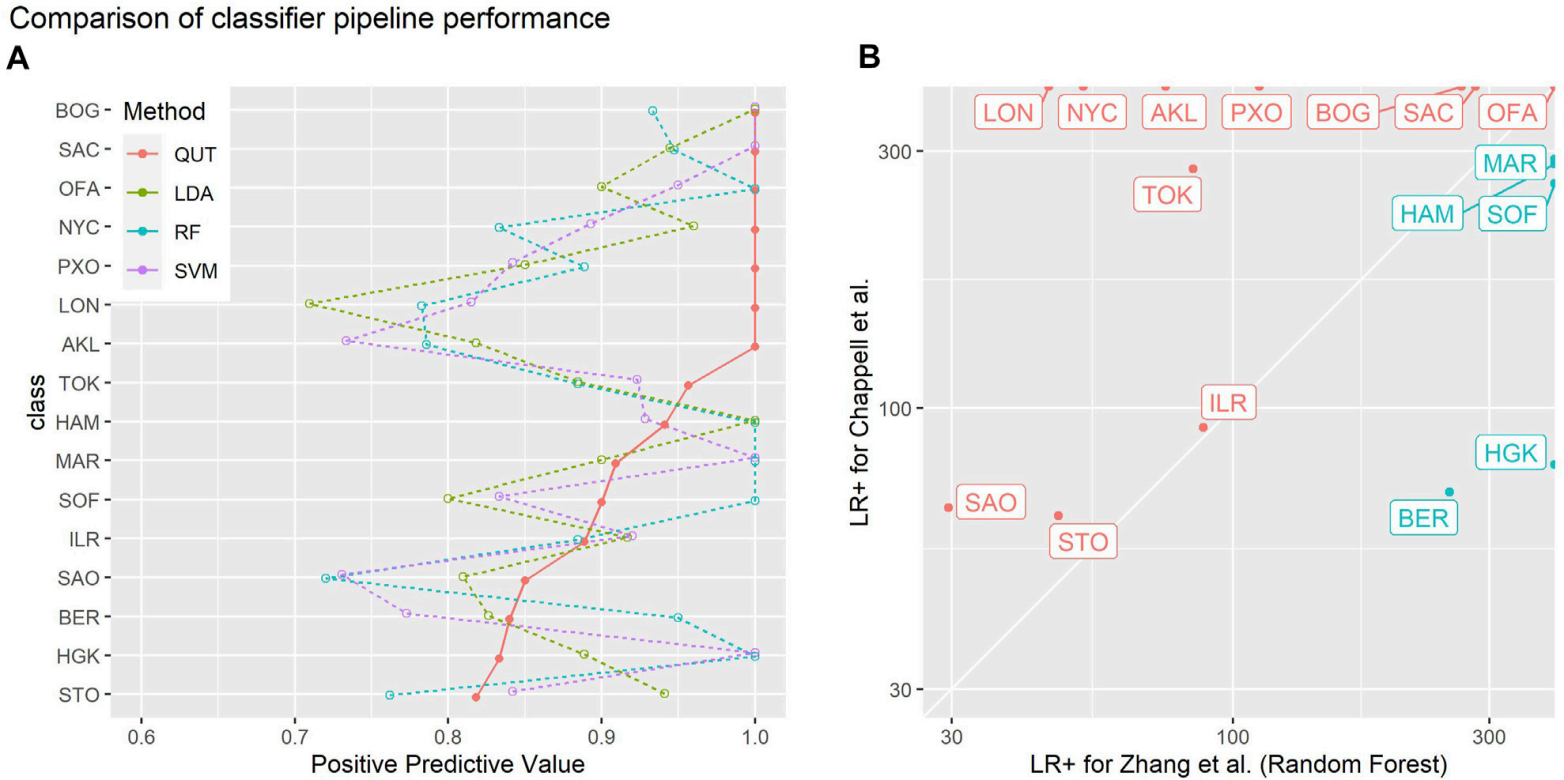
Summaries of the empirical leave-one-out-cross-validation performances of different classifier pipelines on the 2019 CAMDA challenge data used by Zhang et al. (2021): “QUT” refers to the methods of Chappell et al. (2018) (this paper); “LDA”, “RF” and “SVM” denote linear discriminant analysis, random forest, and support vector machine methods evaluated by Zhang et al*.* **(A)** one-versus-all positive predictive values (PPV, a.k.a. precision) for each class. PPV estimates the probability the actual class of an example is X given the classifier predicted it is X: the higher the PPV the better. Classes have been sorted in order of the PPV of the QUT method. **(B)** likelihood ratio of positive outcome (LR_+_) for each class for the method in this paper versus the random forest method of Zhang et al.: the higher the LR_+_, the better that class is correctly discriminated by a classifier. Red points and labels indicate the classes where the method in this paper performed better than random forest method.

Figure 8 visually compares the performance of our method with the linear discriminant analysis, random forest, and support vector machine methods evaluated by Zhang *et al.* on the same 2019 CAMDA challenge data. These plots use the visualisation methods described by Lovell et al. (2021) to present the positive predictive values (true positives/(true positives + false positives), a.k.a. precision) and likelihood ratios of positive outcomes (true positive rate/false positive rate) obtained for each class versus all others. Positive predictive values indicate the probability that an example *predicted* by classifier to belong to class *X* actually belongs to class *X*. The likelihood ratio of a positive outcome reflects the ability of the classifier to correctly classify an instance of a given class.

For most classes, our method performs favourably in comparison to the methods evaluated by Zhang et al. (2021). Figure 8B shows a direct comparison to the random forest method in which our classification pipeline achieves better results in 11 out of 16 classes. While this study is not meant to benchmark the performance of different analysis strategies, these findings suggest that the read signature approach is a useful representation that yields information sufficient to support a competitive classification pipeline.

### 3.4 Run Time Performance

Part of the appeal of k-mer representations is that they are computationally efficient. As the volume of metagenomic data grows, we need analysis methods that can return results in a reasonable time.

Table 1 gives a synopsis of the run times for the most detailed (100 cluster) representation of samples, for k-mers of length 2-5, using an Intel Xeon 8160 with 48 cores. These results demonstrate the computational savings that k-mer representations afford, enabling a new sample of around 1 million reads to be compared against 1065 samples from 23 cities in under 3 min, of which 2 min and 50 s is for paired end merging and filtering out uninformative reads.

**TABLE 1:** Run time performance of different processes in the analysis pipeline. The two steps that involve sequence alignment—paired-end merging and read filtering—dominate the overall run time; the other steps take far less time thanks to the computational efficiency afforded by the k-mer representations they use.

Process	Amount of data	Run time (hours:minutes:seconds)			
Paired-end merging	8,712,731,964 sequences	14:51:04 (avg. 00:00:50 per sample)			
Read filtering		37:22:09 (avg: 00:02:06 per sample) per species			
		2-mers	3-mers	4-mers	5-mers
Signature creation	920,430,590 sequences	00:04:51	00:05:53	00:13:11	00:34:09
Clustering (TVSQ)	100 clusters	00:55:54	01:19:47	03:17:07	07:02:03
Classification	1065 samples, 100 clusters	00:00:02	00:00:07	00:00:34	00:01:54

As Zhang et al. (2021) point out, assigning reads to OTUs is a time consuming step, involving many hundreds of CPU hours for the (smaller) 2019 CAMDA Challenge dataset. Coupled with the classification performances described above, the run times we achieved suggest that k-mer based representations could, at the very least, be useful as a pre-processing filter for more computationally intensive analyses.

## 4 Discussion

The results reported above highlight the effectiveness even of root-level cluster-signatures in characterising the samples, and the utility of ranked retrieval as a lens with which to examine the variations across cluster levels. Moreover, our methods allow very rapid comparison of samples and clustering at scale, making the approach useful even as a preprocessing step prior to more intensive analysis. Nevertheless, there remains some significant work to do in resolving the more complex variations in the misclassified samples. The results obtained with a single cluster per location are both interesting and surprising, achieving accuracy of 49% with the exhaustive 1065-fold LOOXV, and 52% after filtering (Figure 7). This suggests significantly different microbiomes *between* cities, but sufficient similarity within cities to facilitate comparison even with a single signature.

The approach that we took is based on the conjecture that samples from within the same city will tend to have some shared microbial footprint, while those from different cities will tend to have less in common with one other. This approach relies on the availability of representative samples of the microbiome from each city. However, this does not seem to hold true in all cases. We observed that some cities were a lot easier to identify than others. In particular, Barcelona was easy to identify as most samples taken there shared similar signatures, implying a similar microbiome. Hence, left-out samples were easily identified by others left in the collection.

Lisbon on the other hand proved quite difficult to classify and left-out samples did not seem to share the microbial structure of those that remained. Some cities may exhibit a much more diverse microbiome within their boundaries, and many more samples may be required to characterise it properly. Alternatively, there may be different protocols or selections in the samples obtained. This will require further investigation, but for now we cannot assume uniform representation for each city.

Other than in filtering out reads likely to be of human origin, our approach does not attempt to use a reference database, and as such it does not require a comprehensive reference lookup to provide utility.

## 5 Conclusion

We have introduced a novel approach to the Metagenomic Geolocation Challenge based on random projection of the sample reads, demonstrating its potential utility for rapid classification of location-tagged samples. The core advantage of these methods lies in the signature-based representation of each read, which allows faster comparison, clustering and classification of samples and avoids the computationally demanding step of aligning sequences to reference genomes of organisms. k-mer representations might enable us to analyse sequence data a rate that can keep pace with the growth of metagenomic sequence data.

Most importantly we have shown that simple k-mer representations can carry meaningful information about sample origin without the need to construct more elaborate feature vectors. We found that performance generally improved with increasing k-mer length and number of location signatures. Removing uninformative reads also made a material improvement to classification performance.

A number of refinements may be made to the approach: in the nature of the projection, the choice of distance metric and perhaps most notably in the choice of classification method. Yet even using a simple nearest neighbour classifier allowed us to identify at least one same-city sample in the top 10 predictions some 84*%* of the time. Given that there are 1065 such samples available these results appear very convincing: our approach is immediately useful as a pre-processing filter—reserving more accurate and resource hungry methods for the task of distinguishing the most promising candidates—and the results provide a firm basis for more sophisticated extensions.

The main findings of this study are that k-mer representations carry sufficient information to reveal the origin of many of the CAMDA 2020 Challenge metagenomic samples, and that this reference-free approach can be achieved with much less computation than methods that need reads to be assigned to operational taxonomic units. Moreover, there are a number of promising avenues for further research likely to lead to significant improvements in classification accuracy without detracting from the convenience and computational advantages that make these approaches so attractive. These ideas will form the basis for future papers relating to this Challenge.

## Data Availability

The data analyzed in this study is subject to the following licenses/restrictions: Data was made available to participants by the CAMDA challenge organisers. Requests to access these datasets should be directed to CAMDA Challenge: http://camda.info/.
